# Using an integrated knowledge translation approach to build a public health research agenda

**DOI:** 10.1186/1478-4505-12-6

**Published:** 2014-01-29

**Authors:** Anita Kothari, Sandra Regan, Dana Gore, Ruta Valaitis, John Garcia, Heather Manson, Linda O’Mara

**Affiliations:** 1 School of Health Studies, Western University, London, Ontario N6A 5B9, Canada; 2 School of Nursing, Western University, London, Ontario N6A 5C1, Canada; 3 School of Nursing, McMaster University, Hamilton, Ontario L8S 4K1, Canada; 4 School of Public Health and Health Systems, University of Waterloo, Waterloo, Ontario N2L 3G1, Canada; 5 Chronic Disease and Injury Prevention, Public Health Ontario, Toronto, Ontario M5G 1V2, Canada

**Keywords:** Evidence-based Practice, Information dissemination, Research, Public health

## Abstract

**Background:**

Public Health Systems Research is an emerging field of research that is gaining importance in Canada.

**Methods:**

On October 22 and 23, 2012, public health researchers, practitioners, and policy-makers came together at the *Accelerating Public Health Systems Research in Ontario: Building an Agenda* think tank to develop a research agenda for the province.

**Results:**

This agenda included the identification of the six top priorities for research in Ontario: public health performance, evidence-based practice, public health organization and structure, public health human resources, public health infrastructure, and partnerships/linkages.

**Conclusions:**

This paper explores the priorities in detail and hopes to bring more attention to this area of research.

## Background

The public health (PH) sector has made substantial gains in population health and longevity over the past century. Key accomplishments can be found in the areas of vaccination coverage, control of infectious diseases, and recognition of tobacco use as a health hazard, among others [1-4]. However, over the past decade new and persistent health risks such as resurgent infectious diseases, threats of bioterrorism, large-scale natural disasters, and the advance of chronic diseases have threatened and continue to pose risks for the general population [5-9]. These complex PH issues have been described as ‘wicked problems’ – issues with multiple causes requiring a systems approach to effectively address them [10,11]. Reports have focused on the importance of a strong PH infrastructure, backed by a solid evidence base, to respond to these threats [8,12,13]. It is a critical time to focus on public health systems research (PHSR), which can point the way to stronger and more effective mechanisms for preventing, detecting, and addressing emerging population health risks [5,7].

PHSR is a relatively new field of research that has gained momentum in the last decade. In 2003, Mays et al. defined the area as “a field of study that examines the organization, financing, and delivery of public health services within communities, and the impact of these services on public health” [5, p. 180], and the definition has since been updated to emphasize the link between PHSR and health services research [14]. To date, the bulk of PHSR research has been conducted in the United States (US) [15], although research is also originating in countries such as Australia, the United Kingdom, and Canada. Because of differences in conceptualizations of PH, organizational structures and region-specific health issues, no two countries have identical programs of research.

The US has been an international leader in PHSR thanks, in part, to strong encouragement from the Institute of Medicine, sustained funding from the Robert Wood Johnson Foundation, and the establishment of a research network dedicated exclusively to PHSR – the Academy Health PHSR Interest Group [15]. In 2003, the Centers for Disease Control held a meeting with researchers and national partners in order to establish a consensus-based research agenda for PHSR in the US; this agenda has been used to focus the scope of PHSR, identify opportunities for collaboration, leverage research funds, and increase awareness of the field [8]. Subsequent developments and initiatives, such as the establishment of the National Coordinating Center for Public Health Services and Systems Research and authorization of PHSR funding under the Patient Protection and Patient Care Act, allowed for an influx of funding for PHSR that prompted the creation and publication of an updated research agenda in 2012 [14].

In Canada, the process of setting a research agenda has just begun; in May 2011 the Renewal of Public Health Systems research team, comprised of researchers and knowledge users from British Columbia and Ontario (http://www.uvic.ca/research/groups/cphfri/projects/currentprojects/rephs/index.php), held a National Think Tank in order to establish a pan-Canadian Public Health Systems and Services Research agenda. Top priorities that emerged from this Think Tank included data development/PH information systems, PH system performance and governance, and system/organizational structures [16].

One conclusion that emerged from the national Think Tank related to the importance of establishing provincial-level PHSR agendas. Ontario is unique in its PH organizational structure; it is the only province that organizes PH services by grouped municipalities rather than by region. The Health Promotion and Protection Act [17] mandates the 36 PH units in the province to deliver programs and services under direction of local Boards of Health. Minimum requirements for service delivery are outlined in the Ontario Public Health Standards [18]. Because of Ontario’s unique nature in the structure and organization of PH services, the province would benefit from tailored and contextually sensitive PHSR priorities.

With this in mind, Ontario researchers sought funding for a provincial think tank to develop a PHSR research agenda. An integrated knowledge translation (IKT) philosophy was embraced from the start [19]; IKT engages both researchers and decision-makers (i.e., practitioners and policy-makers) in the entire research process – from definition of research questions to implementation of recommendations from findings. It is meant to be collaborative and transformative, and to cross disciplinary boundaries [20]. Ultimately, research questions become more sensitive to policy and practice needs and realities, leading to relevant research that facilitates the uptake of research findings. The core team thus evolved to include researchers, practitioners, and policy-makers from institutions and organizations across the province.

## Methods

The purpose of the *Accelerating Public Health Systems Research in Ontario: Building an Agenda* Think Tank (hereafter referred as ‘the Think Tank’) was to bring together a group of key stakeholders from across Ontario with an interest and expertise in PHSR, as well as national and international PHSR experts, to engage in discussion and debate about PH systems research priorities at the provincial level. This meeting was designed to move toward consensus on the Ontario PHSR agenda, initiate the development of a five-year plan to advance the agenda, and establish a nascent PHSR network. In preparation, an environmental scan of emerging trends in public health and healthcare was conducted to identify relevant contextual issues for a PHSR agenda. An online survey on PHSR priorities in Ontario was also conducted, with participation from a wide range of PH networks across the province, such as health units, PH researchers, research units, PH organizations, academic programs in health studies/PH, and selected non-governmental organizations. This was an attempt to solicit broad input into the priority-setting process.

The Think Tank took place on October 22 and 23, 2012 at the Public Health Ontario building in Toronto, Ontario. Overall, 39 participants attended the Think Tank, including 2 invited international guests from the US and the UK chosen for their expertise in PHSR, and 37 Canadian researchers, practitioners, and policy makers (see the 22 organizations that were represented in Table 1). Although the national level was represented, the majority of participants were from Ontario.

**Table 1:** List of participating organizations, institutions, ministries, and health units

**Academic institutions (Research)**	**Public Health Agencies (Research, Policy and Practice)**
1. Brock University	17. Public Health Agency of Canada
2. McMaster University	18. Public Health Ontario
3. University of Victoria	
4. University of Waterloo	**Professional Associations (Research and Policy)**
5. Western University	19. Ontario Public Health Association
6. University of Toronto	20. Registered Nurses’ Association of Ontario
**Institutes/Centers (Research and Knowledge Translation)**	**Government (Policy)**
7. National Collaborating Centre for Determinants of Health	21. Ontario Ministry of Health and Long-Term Care
8. Propel Centre for Population Health Impact	**International Guests**
9. Canadian Institutes of Health Research	22. Office for State, Tribal, Local and Territorial Support, Centers for Disease Control and Prevention (US)
**Public Health Units (Practice)**	23. Scottish Collaboration for Public Health Research and Policy (UK)
10. Haldimand-Norfolk health unit	
11. Middlesex-London health unit	
12. Ottawa public health	
13. Peel public health	
14. Porcupine health unit	
15. Sudbury & district health unit	
16. Toronto public health	

An external facilitator guided the Think Tank process throughout the two days using a tool called Group Decision Support Software, which allows for idea generation, discussion, and voting by all participants. Instead of conventional brainstorming where ideas are put out by group members and written down on a flipchart, people enter their ideas onto individual laptops, which are then presented for discussion and refinement on a large screen. Participants can also vote to prioritize the ideas using their laptops, and the software ranks them based on the number of votes received. Results are immediately visible on the screen, allowing for a transparent process.

The Think Tank began with a group-brainstorming session to identify reasons for developing a PHSR agenda. The group was presented with the results from the environmental scan and on-line survey, as well as presentations from national and international experts on PHSR, followed by a panel discussion by leaders in the Ontario PH system. Fifteen priorities were presented, compiled in advance by the core Think Tank research team based on priorities generated for the National Think Tank. The group added several more but, after some discussion and clarification, agreed on one distinct new priority: “The role of ‘public’ in PH: community engagement, relationships with key stakeholders, leadership, etc.”

**Figure 1:**
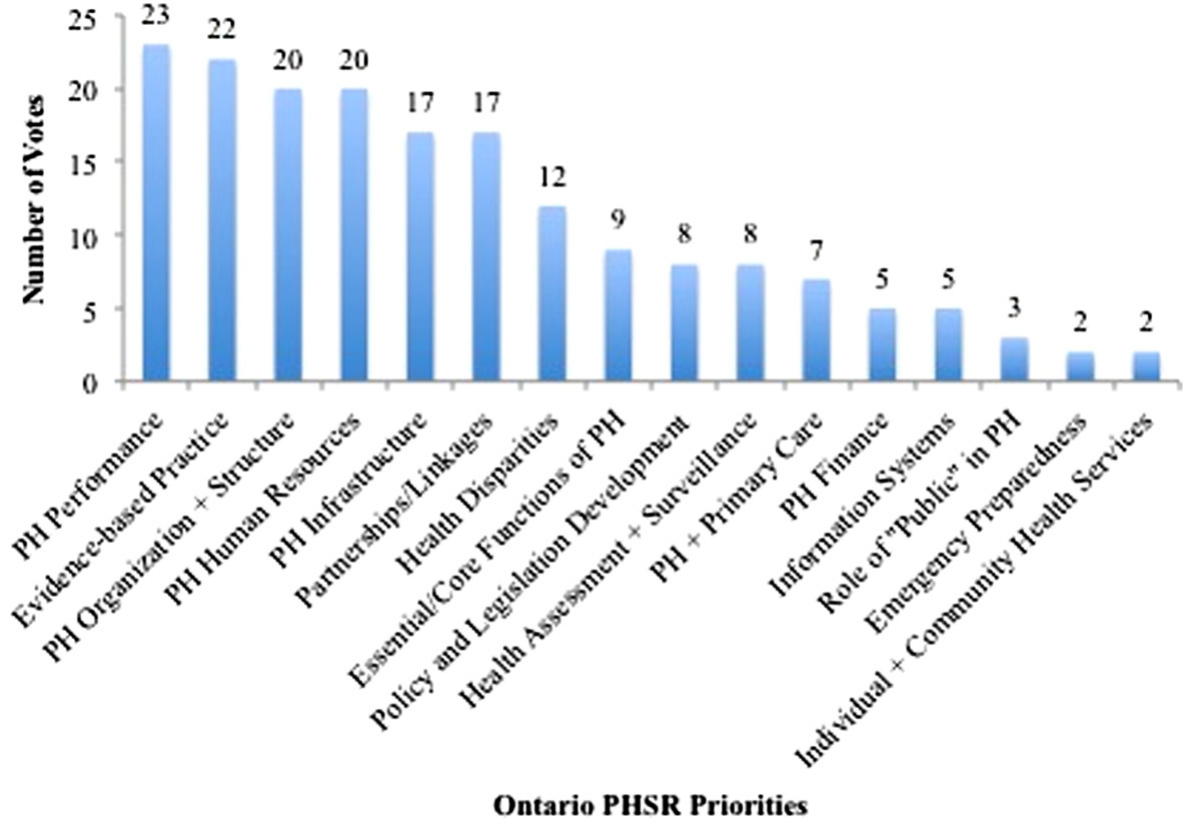
Vote for the top six PHSR priorities for Ontario.

The group then voted and selected the most important six of these (Figure 1). For each priority, participants worked in subgroups to identify potential research questions, methods, and stakeholders who could be important actors in that area. The larger group also highlighted missing partners and next steps required to move the agenda forward. A second panel provided perspectives from leaders about how to effectively build synergies for a PHSR agenda given Ontario’s current context. Finally, participants indicated expressions of interest in and/or commitment to specific priorities. The day concluded with closing remarks and a brief Think Tank evaluation survey.

## Results

Participants collaboratively generated research questions for the top six priorities, described below. For a summary of research priorities and questions, see Table 2.

**Table 2:** Summary of research agenda top priorities and research questions

**Priority**	**Research Questions/sub-themes**
Public	1. How do we develop a conceptual model for the development of indicators?
Health	2. What are the impacts of quality improvement systems on PH system performance?
Performance	3. How do we ensure relevance and feasibility of performance management systems (including data collection) to practitioners? What are various quality improvement initiatives that exist and which are relevant for PH? How do we differentiate workforce performance and outcome of services, &/or outcomes of partnerships? What are various quality improvement initiatives that exist and which are relevant for PH?
	4. How can we track inequities in populations as well as the incorporation of equity into policy and practice?
	5. How cost-effective and cost-efficient is the PH system in ON?
	6. How do you do contribution analysis in PH? What exactly is the attribution/value add/impact of PH?
Evidence-based Practice	7. Who uses evidence? How is evidence used at PHUs and what are the contributing factors (enablers/barriers to using evidence)?
	8. What evidence is used and what is the quality of the data/evidence being used? What exactly are the most effective evidence-based practices?
	9. How do Health Units successfully incorporate evidence informed decision making throughout the health unit? At all levels of the organization?
Public Health Organization & Structure	10. What is the relationship between the way PH is organized (structure, budget, authority, decision-making capacity, mandate) and PH performance (i.e. effective PH practice)?
	11. Are there international “best practice” governance models that result in better PH performance? How to we make inter-jurisdictional comparisons, within ON, between ON and other jurisdictions, and internationally?
	12. Are there preferred organizational models that allow for intersectoral collaboration?
Public Health Human Resources	13. Are the core competencies being used? Where and to what effect? What core competencies are required in the workforce to take action on the social determinants of health? What common skills to do we all need? Do PH practitioners have the necessary competencies to implement the OPHS effectively? How do the competencies of existing PH workforce map onto the national competencies? What core competencies should be integrated into university undergraduate (i.e. nursing, health sciences, inspectors, social work, dental, nutrition, medicine) and masters programs to ensure an adequately prepared workforce?
	14. Is there surge capacity in PH?
	15. What are the types, numbers and distribution of (various) PH professionals across ON, including workforce-to-population ratios (which may be adjusted for population health status, geography, diversity and other factors)? What are the best models of forecasting the PH workforce needs - and what factors would be integrated in to this model?
Public Health Infrastructure	16. What is the population size, geography required to achieve ‘critical mass’ (consider adaptability)? Is there an ideal population size for each PH unit (matching the PH infrastructure to the population)?
	17. How can access to and use of information, data and evidence informed practice improve practice?
	18. How does access to a full range (local and otherwise) of data about health and its determinants impact practice?
	19. What components (e.g., skill mix of the workforce, finances) of the PH infrastructure are most influential? And how do they influence each other? How do they vary across the province?
Partnerships & Linkages	20. How do we assess the meaning, value and outcome of our partnerships from multiple perspectives - including our partners (for example in PH policy, programs and practice)? What are the best indicators - how do we know/measure effective partnerships and partnership processes?
	21. What are the factors enabling/hindering effective partnerships at different levels (e.g. institution, community, etc.)? What are the processes to enable multi-level partnerships, and outcomes associated with these?
	22. Building partnerships across different entities: How do we build partnerships between service, policy and research? What is the most effective/appropriate way to partner with industry? Should PH partner with industry? How does PH partner with the LHINs? What are the linkages? How much time, financial resources, etc should be invested (from all parties) in creating and maintaining partnerships? Are we engaging the right partners for intersectoral action? How do we create and mobilize partnership across PHUs? Where should PH partner with private sector?
	23. What is the role of PH as a convener and steward of partnerships across community organizations with health and among other sectors? What are the infrastructure requirements to enable this to occur?
	23. How can we use partnerships effectively to reach equitable service delivery and ultimately health equity?

### Priority #1: Public Health Performance

The PH Performance priority was concerned with creating theoretically-based performance indicators and measurement tools to evaluate the effectiveness, efficiency, equity of access, and impact of PH services, with a view towards improving quality and safety. Some examples of research questions that were brainstormed were: What quality improvement initiatives exist and what are their impacts on PH system performance? How do we create a conceptual model for the development of indicators? How do we ensure relevance and feasibility of performance management systems (including data collection) to practitioners? How can we track inequities in populations as well as the incorporation of equity into policy and practice?

### Priority #2: Evidence-Based Practice

Evidence-based Practice focused on the challenge of using existing evidence appropriately to guide PH services and decision-making processes, and inform future research initiatives. The group identified the following research questions: Who uses evidence, how is it used at PH units and what are the enablers/barriers to using it? What is the quality of the data/evidence being used? What exactly are the most effective evidence-based practices? How do health units successfully incorporate evidence informed decision making at all levels of the health unit?

### Priority #3: Public Health Organization & Structure

The PH Organization and Structure priority examined how the size, boundaries, and structures of PH agencies/departments impact the delivery and performance of PH services. Some of the research questions relevant for researchers, practitioners, and policy-makers were: What is the relationship between the way PH is organized (structure, budget, authority, decision-making capacity, mandate) and PH performance? Are there international “best practice” governance models that result in better PH performance? Are there preferred organizational models that allow for intersectoral collaboration?

### Priority #4: Public Health Human Resources

The PH Human Resources priority focused on how to recruit and retain PH professionals, while addressing the issues of education and accreditation. Participants highlighted the importance of the PH workforce in every PHSR priority, but key questions centered on the core competencies, for example: Are the core competencies being used? Where and to what effect? What are the core competencies required in the workforce to take action on the social determinants of health? Do PH practitioners have the necessary competencies to implement the Ontario Public Health Standards effectively? Questions about surge capacity in PH and the types, numbers and distributions of PH professionals across Ontario were also asked.

### Priority #5: Public Health Infrastructure

The PH Infrastructure priority was concerned with ensuring that the necessary infrastructure resources (organizational structures, financing systems, workforce characteristics, and delivery mechanisms and technology) are in place to implement effective and appropriate interventions for individuals and communities, as well as at the provincial and national levels. Important research questions relating to infrastructure were listed as: What is the population size, geography required to achieve ‘critical mass’? Is there an ideal population size for each PHU (matching the public health infrastructure to the population)? What components (e.g., skill mix of the workforce, finances) of PH infrastructure are most influential and how do they influence each other across the province?

### Priority #6: Partnerships and Linkages

The last priority, Partnerships and Linkages, is related to creating and mobilizing partnerships/linkages to improve PH system performance (i.e., within and between government, PH agencies, community-based organizations, health care providers, educational institutions, and private sector organizations). Research questions focused on assessing the meaning, values and outcomes of partnerships, as well as techniques for partnership building across different sectors and evaluating partnership outcomes. Some examples of questions were: What are the factors enabling/hindering effective partnerships at different levels (e.g., institution, community, etc.)? What is the role of PH as a convener and steward of partnerships across community organizations with health and among other sectors? How can we use partnerships effectively to reach equitable service delivery and ultimately health equity?

A brief evaluation survey was conducted with participants several months after the Think Tank (May 2013) to understand the short-term impacts of the conference. Sixteen out of 36 invited participants responded, leading to a response rate of 44.4%. This level of participation was expected given the many priorities juggled by these high-level stakeholders. Although the survey was anonymous and it was not possible to identify who exactly participated, the demographic information showed that a mix of policy-makers, researchers, and practitioners were represented in the responses. Respondents noted that they became more familiar with PHSR as a result of the Think Tank (on a scale of 1–10, participants rated their knowledge of PHSR as 6.69 on average before the Think Tank and 8.26 after the Think Tank). Respondents also prioritized PHSR more as a result of the Think Tank (on a scale of 1–10, participants rated the importance of PHSR as 6.3 on average before the Think Tank and 7.9 after the Think Tank). A full 40% of respondents gave the highest possible ranking (10) for the importance of PHSR. Further, 58.3% of respondents indicated that they developed new professional relationships as a result of the Think Tank, 25% formed new partnerships, 41.7% participated in research proposals related to the PHSR agenda that came out of the Think Tank, 75% advocated for or encouraged attention to PHSR priority areas in their professional practice, 33% became more immersed in PHSR literature, and 16.7% participated in program development related to the PHSR agenda.

## Discussion

### Comparison with the US agenda

The priorities generated by the Ontario Think Tank had some similarities and differences with the most recent research agenda generated in the US. While there is some overlap between the top six Ontario priorities and the US’ broad domains of PH Workforce, PH Structure and Performance, PH Financing, and PH Information and Technology, the US agenda contained a total of 72 research questions that were split into 14 thematic areas [21]. While some Ontario priorities (e.g., PH workforce, PH performance) matched with US domains, other Ontario priorities (e.g., partnerships) matched with thematic areas. Some apparent differences are due to the terminology used – for example, key questions in ‘evidence-based practice’ on the Ontario agenda line up in many ways with the US’ ‘PH Information and Technology’ domain. Other priorities that did not make it into ON’s top six (e.g., PH financing) are highlighted in the US agenda. The multitude of thematic areas and research questions in the US compared to Ontario is not surprising given the diversity of structures and organizations that make up the US’s PH systems, compared to Ontario’s fairly centralized PH system. For example, funding for PH in the US can come from a variety of sources and differs between and within states. Limited financial resources have been recognized as a significant challenge for US PH systems [22]. Ontario, on the other hand, has experienced an investment in financial resources for PH, partly as a result of PH reform following emergencies such as SARS (although this infusion of funds has not extended to research funding).

In the US PHSR agenda, the social determinants of health and health disparities were categorized as a thematic area within PH performance and structure. In Ontario, the priority ‘health disparities’ was ranked as a top priority on the online survey and discussed as an extremely important topic in the Think Tank; however, it failed to be included in the top six priorities. This may have been due to a discussion during the Think Tank to remove ‘health inequities’ as an individual priority because addressing health inequities was assumed to be integral to PH (i.e., all priorities) by participants. As such, the agreement on the importance of health inequities may not be immediately apparent in the choice of the top priorities for those who were not involved in the discussion.

### Think Tank goals vs. outcomes

This integrated knowledge translation process was seen as a first step in a collaborative research program. While not all participants will become part of future research project teams under each thematic area, the collaborative process of identifying relevant priorities from the perspective of researchers, practitioners, and policy-makers will hopefully pave a smooth path for those with the capacity to take part in research. The Think Tank had a measurable impact on the knowledge, attitudes, and practices of those who attended.

The aims to build connections between individuals and organizations in this field, bring more attention and awareness to PHSR, and build consensus on Ontario’s PHSR agenda, particularly in the priority areas identified in the Think Tank, were achieved. The core Think Tank team is also in the process of creating an Ontario PHSR website and reaching out to additional stakeholders to expand and diversify a budding PHSR network. While Think Tank participants brainstormed steps in further developing the research agenda (including identifying a steward or convener for the agenda, creating an inventory of knowledge around PHSR, refining and prioritizing research questions, and aligning questions with key issues for policy and decision-makers), currently there is not sufficient infrastructure to develop and implement a concrete 5-year plan. In order to reach its full potential in Ontario, PHSR needs to be acknowledged and prioritized in funding opportunities. The US has been able to achieve significant advances in this field due to sustained financial support from the Robert Wood Johnson Foundation and dedicated funding. In Canada, while PHSR research can be integrated with mainstream health services research, there are no dedicated requests for proposals from our national funding body. The authors recommend that the Canadian Institutes of Health Research put out a strategic call for PHSR proposals, so as not to lose the opportunity to systematically strengthen PHSR in the province.

### Limitations

There were some limitations to the Think Tank agenda-setting process. Although efforts were made to invite a diverse range of key players with a stake in PH in Ontario, it was recognized at the meeting that some important stakeholders were not represented in the room (Table 3). Community representation was lacking as well as representation from other relevant sectors (e.g., education). The priorities on the agenda may have been influenced by the distribution of organizations represented at the Think Tank. For example, if representatives from the acute health care sector or organizations advocating around specific diseases had been present (e.g., hospitals, Heart and Stroke Foundation, Cancer Care Ontario, etc.), the agenda may have been more focused around the burden of particular illnesses. If allied sectors had been present (e.g., education, social services), the social determinants of health may have come more to the forefront on the agenda. The list of ‘missing stakeholders’ compiled by participants towards the end of the meeting will be used in future efforts to broaden and expand the PHSR agenda.

**Table 3:** List of missing stakeholders from the agenda-setting process

**Wider Community**	**Acute Health Care**
• Citizens/The public	• Hospitals
• Aboriginal groups	**Institutes/Centers**
• Representatives of community groups	• National Collaborating Centers for Public Health (beyond the National Collaborating Centre for Determinants of Health)
**Professional Associations/Networks**	• Institute for Clinical Evaluative Sciences
• Association of Nursing Directors and Supervisors of Ontario Official Health Agencies	• Wellesley Institute
• Association of Public Health Epidemiologists in Ontario	• Caledon Institute of Social Policy
• The Board of Health section of the Association of Local Public Health Agencies	• Funders (beyond the Canadian Institutes of Health Research)
• Association of Municipalities of Ontario	• Institut National de Santé Publique du Québec
• Canadian Health Human Resources Network	• BC Centre for Disease Control
• Ontario Health Human Resources Network	**Government Partners**
• Ontario Society of Nutrition Professionals in Public Health	• First Nations and Inuit Health Branch
• Health Promotion Ontario	• Ontario Ministry of Children and Youth Services
• Association of Ontario Health Centres	• Ontario Ministry of Education
• Association of Family Health Teams of Ontario	• Municipal government (e.g., school boards)
**Public Health Agencies/Organizations**	**Education Sector**
• Heart and Stroke Foundation of Ontario	• All schools and programs that offer public health degrees
• Canadian Cancer Society • Canadian Partnership Against Cancer	• Health professional programs (e.g., Schools of Nursing, Medicine, Nutrition, etc.)

Another limitation lies in the time constraints inherent in a 2-day Think Tank. The process that led to the selection of the top six priorities was based on group brainstorming followed by voting. The time limitations placed upon the group precluded an exhaustive discussion and a completely rigorous voting method. Although participants were provided with criteria upon which to base their decisions (PHSR-related, collaborative, urgent, comparable across jurisdictions, and intervention-friendly), criteria may have been weighted differently by different participants. Efforts were made, however, to ensure that everyone’s voice was heard in the discussion and voting process, which was enabled through Group Decision Support Software.

Despite these limitations, the majority of participants were very satisfied with the Think Tank and felt committed to the agenda that was developed, based on the evaluation results. Some strengths include international input, key players knowledgeable about the Ontario context, stakeholders from the previous National Think Tank, and efficient generation of ideas through an expert facilitator, all of which helped to result in the accomplishment of the meeting objectives.

## Conclusions

In conclusion, the Think Tank was meant to bring together key stakeholders from research, policy, and practice to develop a structured agenda for PHSR in Ontario, identify partners and research methods for top priorities, and build momentum for the next steps forward. The process of consensus-building to create a collective program of research has successfully advanced PHSR agendas in the US, British Columbia, and now Ontario. The authors can recommend this process with confidence to other regions and countries looking to strengthen their PHSR agendas, provided efforts are made to include representation of all important actors in the given PH system. It is hoped that the publication of the Ontario PHSR priorities will spark interest, bring focus, and lead to collaborative inquiries in this important area of research.

## Abbreviations

PH: Public health; PHSR: Public Health Systems Research; IKT: Integrated Knowledge Translation; ON: Ontario; US: United States.

## Competing interests

The authors declare that they have no competing interests.

## Authors’ contributions

All authors contributed equally to this work. All authors led the conceptualization, implementation and analysis of the Think Tank. All authors also contributed to the writing of the manuscript and read and approved the final version.
